# A conversation on the impacts and mitigation of air pollution

**DOI:** 10.1038/s41467-021-25401-0

**Published:** 2021-10-04

## Abstract

Air pollution and the associated health impacts affect millions of people around the world. In this Q&A, Professor Haikun Wang, an expert on the health risks of air pollution and climate change at Nanjing University, shares with *Nature Communications* their thoughts on the impacts of air pollution and the policies needed to tackle emissions.

**Figure:**
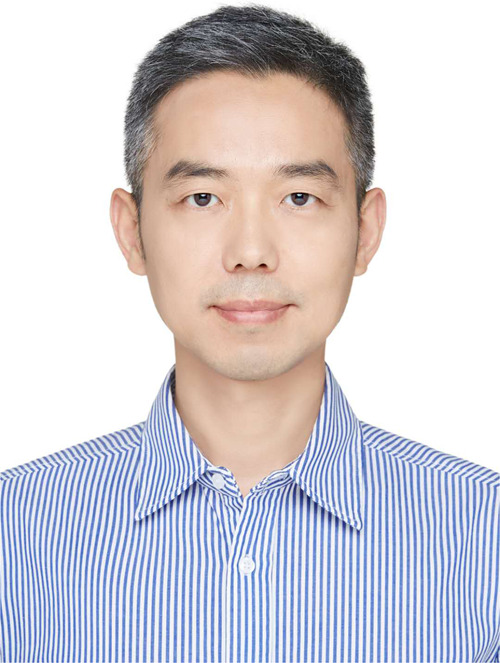
Haikun Wang

1. What aspect of air pollution concerns you the most?

My primary concern is the impacts, especially the health impacts of air pollution and their socioeconomic drivers, such as trade, population aging, income and etc. Air pollution has become a leading environmental risk factor affecting urban and rural populations around the world. The Global Burden of Diseases Study estimates that ambient (outdoor) air pollution of particulate matter and ozone is responsible for nearly 6.7 million premature deaths worldwide in 2019. And the majority of these deaths occurred in developing countries with large populations and serious air pollution such as India and China. The health impacts of air pollution are not only affected by the exposure level but are also closely related to the exposed population, social and economic factors, which therefore must be considered in order to formulate effective air pollution control policies to protect human health.

2. What are your thoughts on current policy enforcement, and how well or not this is being achieved?

Substantial health benefits have been achieved around the world through implementing air pollution control policies during the last several decades. For example, the USA Clean Air Act was implemented in the 1970s, and the resulting cleaner air in 2020 has been estimated to prevent ~230,000 deaths. The historical control policies on vehicle emissions in China from 1998 to 2015 have also led to substantial reductions in air pollution impacts - the number of deaths attributable to air pollution in 2015 would have been around 510,000 higher than without those controls. More recently, since the promulgation in 2013 of China’s toughest-ever Air Pollution Prevention and Control Action Plan, the PM_2.5_ concentration has substantially declined, by around 30% in 2017.

However, there are still major challenges ahead. Developed countries with cleaner air should continue to reduce pollution, because recent epidemiological studies have found that reducing PM_2.5_ concentrations further from an already low level would bring much greater health benefits that might cover their cost of pollution control policies. For developing countries, they might bear the impact of pollution transfer from developed countries via outsourcing and international trade, but equally their pollution might also affect other regions through long-range transboundary transportation. The capacity of supporting scientific decision-making, supervision and management also needs to be strengthened to ensure the full implementation of air pollution control policies in developing countries. This highlights the requirement for successful collaboration between scientists, engineers, and policy makers from regional to global scales to develop cost-effective technologies and policies to address these challenges.

3. How effective is voluntary action vs government mandated policy in reducing air pollution?

Scholars and policy makers have debated the effectiveness of voluntary action and government mandated policies in mitigating environmental pollution for many years. I personally think that mandatory powers of government are currently more effective in reducing air pollution. Firms and citizens usually maximize their self-interest but lack the motivation to pay extra money to mitigate air pollution, because the costs of environmental pollution (e.g., negative health impacts of air pollution) are usually not fully evaluated or included in their cost (a.k.a. indirect cost). It could be problematic to expect too much from voluntary actions to reduce air pollution. Of course, mandated policies also have limitations. For example, they might have negative implications on the competitiveness of firms such as causing reduced profits margins. Mandated policies might also be easily influenced by the decisions of individual governments, such as President Trump’s withdrawal from the Paris Agreement. From this perspective, we need to study how to combine the voluntary action with mandated policy more effectively in the future, especially as the public awareness for environmental protection is generally increasing with social-economic development.

4. Socioeconomic factors such as income, education and wealth have been shown to play a key role in public health air pollution impacts. What needs to be done to ensure that policies developed are equitable and just?

Socioeconomic factors could affect air pollution and related health burdens, not only within a country, but also across various countries through trade. Recently, air pollutant emission levels have grown rapidly in some developing countries but stabilized or even decreased in many developed countries. This is partly because developing countries produce and export emission-intensive products to support the consumption in developed countries. Of course, such trade would increase economic efficiency and benefit both developed and developing countries. However, the environmental costs of developing countries are much higher than that of developed countries relative to their respective economic gains. Developing countries experience air pollution exposure inequality through global trade. Similar problems might also exist in domestic trade between developed and developing regions within a country like China.

Such environmental inequalities reflect the different economic development stages of trading partners. Developing countries or regions are often not able to afford technological innovations needed to mitigate pollution. Thus, establishing an effective collaborative framework between developed and developing countries to technologically and financially support pollution control and R&D efforts in developing countries would help to improve the overall quality of their exports while mitigating regional inequality. Additionally, a compensation scheme based on the principle “who benefits, who compensates” may be a practical solution to allocate the ecological burdens equally among countries or regions.

5. Technological advances to mitigate air pollution such as retrofitting coal-fired plants are touted as potential cost-effective solutions. What are the most promising recent advances to mitigate against pollutants?

As fossil fuels are still the major energy source supporting the global economic development, existing coal-fired power plants and fuel vehicles can not be replaced instantly. The traditional end-of-pipe pollution control technologies, or process control technologies, are still very important for air pollution control, especially in some developing countries such as China and India. In the short term, technological advances such as retrofitting coal-fired plants might be cost-effective solutions. However, as more aggressive pollution controls tend to cost more money, the marginal abatement cost usually go up rapidly with emission levels getting lower. This has already happened in some developed countries and also in China’s developed regions like Shanghai. From a technical point of view, clean and low-carbon energy, such as wind and solar energy, should be the ultimate solution to the energy needs and air pollution in the future. Especially in the context of addressing global climate change, application of the low-carbon energies would bring the synergistic effect of reducing CO_2_, CH_4_ and other greenhouse gas emissions. Researches have illustrated that renewable energies are more cost-effective compared to traditional fossil fuels if we consider the cost of their impacts (e.g., health impacts).

6. Do you hold out more hope for technological solutions, or political action, as a means to reduce air pollution?

In my opinion, they are equally important. On the one hand, technological solutions are the foundation, which provides us with the basic tools for air pollution control. Good policies and management, on the other hand, can not only accelerate the application of advanced technologies but also make these tools work more efficiently. Developed countries usually have relatively higher social and economic management efficiency. They should focus more on the development of new technologies to achieve further reductions in air pollution, and provide technological support for developing countries. Policy measures should also be strengthened to mitigate direct and indirect pollution transfer accompanied by the outsourcing of emission-intensive industries from developed countries to developing ones. For developing countries, they need to strengthen their political determination to protect the environment and public health, and minimize air pollution while the economy is growing. At the same time, they should learn and adopt advanced technologies and management experiences from developed countries, and maximize the effect of their existing technological solutions.

7. Finally, how would you like collaboration between physical, health and policy scientists working on air pollution to improve?

Air pollution, by definition, includes the emission, transformation, impact, and mitigation of multiple air pollutants, which involves physical, health, and policy sciences. Therefore, we should, at first, understand that a robust collaboration between scientists from these fields is crucial to successfully address air pollution issues. For example, if one has a research issue/objective to evaluate the health effects of a specific air pollution policy, one might need help from other natural and/or social scientists. A comprehensive study on air pollution is usually impossible to be completed by one individual scientist or the scientific community only, and cooperation with the public, policy makers, and even private corporations are sometimes necessary.

Second, the effective cooperation between scientists from different fields (often with different ideologies, methods and tools) is challenging. Sufficient communication with colleagues of different knowledge backgrounds in atmospheric science, public health, and policy analysis is essential. It would help you to understand the tools and data each scientist can bring, and to connect these data and tools effectively across disciplines. Only with this, the technical roadmap and detailed approaches (e.g., including policy scenario analysis–emissions–atmospheric transport–health effects–cost/benefit) can be determined for a study on air pollution policy.

Third, we should also keep in mind that such research that integrates physical, medical, and social sciences on air pollution might never be perfect. But they are the effective (if not the only) solutions to comprehensive issues like air pollution, and the results would become more reliable with the improvement in the individual fields and the collaboration among physical, health and policy scientists.

Finally, data and results should be interpreted and shared by collaborators to keep transparency. Clear communication of research results and their uncertainty with the public and policy makers is also a must. I believe that, with robust scientific results and transparent policy-making process, we can ultimately design and implement more efficient air pollution policies.

